# Correction: Risk factors for post-induction hypotension in patients with end-stage renal disease undergoing general anesthesia

**DOI:** 10.1371/journal.pone.0356297

**Published:** 2026-08-17

**Authors:** Supachai Trikisayaveach, Laortip Rattanapittayaporn, Jatuporn Pakpirom, Chutida Sungworawongpana, Suttasinee Petsakul

There are errors in the author affiliations. The correct affiliations are as follows,

Supachai Trikisayaveach^1^, Laortip Rattanapittayaporn^1^, Jatuporn Pakpirom^1^, Chutida Sungworawongpana^1^, Suttasinee Petsakul^1^

**1** Department of Anesthesiology, Faculty of Medicine, Prince of Songkla University, Hat Yai, Songkhla, Thailand.
