# Supplementary material for: Risk factors for post-induction hypotension in patients with end-stage renal disease undergoing general anesthesia
Source: PLoS One. 2026 May 29;21(5):e0350083. doi: 10.1371/journal.pone.0350083 (PMC13221025; doi:10.1371/journal.pone.0350083)

PLOS ONE

Risk factors for post-induction hypotension in patients with ESRD undergoing general anesthesia

--Manuscript Draft--

**Manuscript Number:**	PONE-D-25-50403
**Article Type:**	Research Article
**Full Title:**	Risk factors for post-induction hypotension in patients with ESRD undergoing general anesthesia
**Short Title:**	Risk factors for post-induction hypotension in ESRD
**Corresponding Author:**	Laortip Rattanapittayaporn<br>Prince of Songkla University Faculty of Medicine<br>Hatyai, Songkhla THAILAND
**Keywords:**	Post-induction hypotension, patients with ESRD, risk factors, non-cardiac surgery, general anesthesia
**Abstract:**	Abstract<br>Background: Patients undergoing non-cardiac surgery under general anesthesia frequently experience post-induction hypotension. Patients with end-stage renal disease (ESRD) may be highly susceptible to morbidity owing to decreased compensatory vasoconstrictor responses, volume depletion after recent dialysis, and an increased risk of cardiovascular events. Identifying the risk factors of post-induction hypotension in patients with ESRD receiving general anesthesia is crucial.<br>Methods: This retrospective cohort study included 635 patients with ESRD aged > 18 years who underwent non-cardiac surgery using general anesthesia with endotracheal intubation between January 2012 and December 2022. Post-induction hypotension was defined as a 30% decrease in MAP from baseline within 20 minutes of induction and hypotension treatment via vasopressor therapy. Two or more endotracheal intubation attempts, intubation via a double-lumen endobronchial tube, intubation before surgery, vasopressor or inotropic infusion before general anesthesia induction, and incomplete data were excluded from analysis. Univariate analysis was performed using logistic regression analysis, and the associated factors (P < 0.2) were subsequently selected for further multivariate logistic regression analysis. Factors associated with post-induction hypotension in the fitted model were evaluated using receiver operating characteristic (ROC) curves.<br>Results: The median number of dialysis days was 2 day. Older age, especially over 65 years (p <0.001), long-term use of oral anti-diabetic drugs (p = 0.012), increased propofol dosage during induction (p =0.002), rapid sequence induction (p < 0.001), and emergency surgery (p = 0.002), were associated with post-induction hypotension. Differences in the number of days of dialysis or in weight compared to dry weight were not statistically significant. Post-induction hypotension in patients who underwent non-cardiac surgery under general anesthesia was 44.1% (95% CI]: 40.28:47.98).<br>Conclusion: The risk of post-induction hypotension in patients with ESRD undergoing general anesthesia and emergency surgery was high in those agedover 65 years, patients on long-term oral anti-diabetic therapy, and cases involving rapid sequence induction or high doses of propofol.<br>Keywords: Post-induction hypotension, patients with ESRD, risk factors, non-cardiac surgery, general anesthesia
**Order of Authors:**	Laortip Rattanapittayaporn
	Supachai Trikisayaveach
	Jatuporn Pakpirom
	Chutida Sungworawongpana
	Suttasinee Petsakul
**Additional Information:**	

Powered by Editorial Manager® and ProduXion Manager® from Aries Systems Corporation

Question	Response
**Financial Disclosure**<br><br>Enter a financial disclosure statement that describes the sources of funding for the work included in this submission. Review the submission guidelines for detailed requirements. View published research articles from *PLOS ONE* for specific examples.<br><br>This statement is required for submission and **will appear in the published article** if the submission is accepted. Please make sure it is accurate.<br><br>**Funded studies**<br>Enter a statement with the following details:<br>• Initials of the authors who received each award<br>• Grant numbers awarded to each author<br>• The full name of each funder<br>• URL of each funder website<br>• Did the sponsors or funders play any role in the study design, data collection and analysis, decision to publish, or preparation of the manuscript?<br><br>Did you receive funding for this work?	The author(s) received no specific funding for this work.
**Competing Interests**<br><br>Use the instructions below to enter a competing interest statement for this submission. On behalf of all authors, disclose any competing interests that could be perceived to bias this work—acknowledging all financial support and any other relevant financial or non-financial competing interests.<br><br>This statement is required for submission and **will appear in the published article** if the submission is accepted. Please make sure it is accurate and that any funding sources listed in your Funding Information later in the submission form are also	no competing interests exist

declared in your Financial Disclosure statement.<br><br>View published research articles from *PLOS ONE* for specific examples.<br><br>**NO authors have competing interests**<br><br>Enter: *The authors have declared that no competing interests exist.*<br><br>**Authors with competing interests**<br><br>Enter competing interest details beginning with this statement:<br><br>*I have read the journal's policy and the authors of this manuscript have the following competing interests: [insert competing interests here]*<br><br>* typeset	
**Ethics Statement**<br><br>Enter an ethics statement for this submission. This statement is required if the study involved:<br><br>• Human participants<br>• Human specimens or tissue<br>• Vertebrate animals or cephalopods<br>• Vertebrate embryos or tissues<br>• Field research<br><br>Write "N/A" if the submission does not require an ethics statement.<br><br>General guidance is provided below. Consult the submission guidelines for detailed instructions. **Make sure that all information entered here is included in the Methods section of the manuscript.**	The study protocol was reviewed and approved by the Human Research Ethics Committee of the Faculty of Medicine at the Prince of Songkhla University (REC 66-290-8-1). Hospital records were obtained from the hospital information system (HIS), and patient consent was waived by the ethics committee because of the retrospective review of the medical records.

Powered by Editorial Manager® and ProduXion Manager® from Aries Systems Corporation

**Format for specific study types**<br><br>**Human Subject Research (involving human participants and/or tissue)**<br>• Give the name of the institutional review board or ethics committee that approved the study<br>• Include the approval number and/or a statement indicating approval of this research<br>• Indicate the form of consent obtained (written/oral) or the reason that consent was not obtained (e.g. the data were analyzed anonymously)<br><br>**Animal Research (involving vertebrate animals, embryos or tissues)**<br>• Provide the name of the Institutional Animal Care and Use Committee (IACUC) or other relevant ethics board that reviewed the study protocol, and indicate whether they approved this research or granted a formal waiver of ethical approval<br>• Include an approval number if one was obtained<br>• If the study involved *non-human primates*, add *additional details* about animal welfare and steps taken to ameliorate suffering<br>• If anesthesia, euthanasia, or any kind of animal sacrifice is part of the study, include briefly which substances and/or methods were applied<br><br>**Field Research**<br><br>Include the following details if this study involves the collection of plant, animal, or other materials from a natural setting:<br>• Field permit number<br>• Name of the institution or relevant body that granted permission	
**Data Availability**<br><br>Authors are required to make all data underlying the findings described fully available, without restriction, and from the time of publication. PLOS allows rare exceptions to address legal and ethical concerns. See the PLOS Data Policy and FAQ for detailed information.	Yes - all data are fully available without restriction

A Data Availability Statement describing where the data can be found is required at submission. Your answers to this question constitute the Data Availability Statement and **will be published in the article**, if accepted.<br><br>**Important:** Stating 'data available on request from the author' is not sufficient. If your data are only available upon request, select 'No' for the first question and explain your exceptional situation in the text box.<br><br>Do the authors confirm that all data underlying the findings described in their manuscript are fully available without restriction?	
**Describe where the data may be found in full sentences. If you are copying our sample text, replace any instances of XXX with the appropriate details.**<br><br>• If the data are **held or will be held in a public repository**, include URLs, accession numbers or DOIs. If this information will only be available after acceptance, indicate this by ticking the box below. For example: *All XXX files are available from the XXX database (accession number(s) XXX, XXX.)*.<br>• If the data are all contained **within the manuscript and/or Supporting Information files**, enter the following: *All relevant data are within the manuscript and its Supporting Information files.*<br>• If neither of these applies but you are able to provide **details of access elsewhere**, with or without limitations, please do so. For example:<br><br>*Data cannot be shared publicly because of [XXX]. Data are available from the XXX Institutional Data Access / Ethics Committee (contact via XXX) for researchers who meet the criteria for access to confidential data.*<br><br>*The data underlying the results presented in the study are available from (include the name of the third party*	All relevant data are within the manuscript and its Supporting Information files.

and contact information or URL).<br>• This text is appropriate if the data are owned by a third party and authors do not have permission to share the data.<br><br>* typeset	
Additional data availability information:	Tick here if the URLs/accession numbers/DOIs will be available only after acceptance of the manuscript for publication so that we can ensure their inclusion before publication.; Tick here if your circumstances are not covered by the questions above and you need the journal's help to make your data available.

Powered by Editorial Manager® and ProduXion Manager® from Aries Systems Corporation

Click here to access/download;Manuscript;manuscript_ESRD_edit.docx

Risk factors for post-induction hypotension in patients with ESRD undergoing general anesthesia

Supachai Trikisayaveach[1], Laortip Rattanapittayaporn[2]*, Jatuporn Pakpirom[2], Chutida Sungworawongpana[2], Suttasinee Petsakul[2]

[1] Department of Anesthesiology, Somdech Phra Pinklao Hospital, Bangkok, Thailand.

[2] Department of anesthesiology, Prince of Songkla University, Hatyai, Songkhla, Thailand.

Short title: Risk factors for post-induction hypotension in ESRD

*** Corresponding author**:

E-mail: laortip.r@gmail.com (LR)

Word count: 4059

Abstract

Background: Patients undergoing non-cardiac surgery under general anesthesia frequently experience post-induction hypotension. Patients with end-stage renal disease (ESRD) may be highly susceptible to morbidity owing to decreased compensatory vasoconstrictor responses, volume depletion after recent dialysis, and an increased risk of cardiovascular events. Identifying the risk factors of post-induction hypotension in patients with ESRD receiving general anesthesia is crucial.

Methods: This retrospective cohort study included 635 patients with ESRD aged > 18 years who underwent non-cardiac surgery using general anesthesia with endotracheal intubation between January 2012 and December 2022. Post-induction hypotension was defined as a 30% decrease in MAP from baseline within 20 minutes of induction and hypotension treatment via vasopressor therapy. Two or more endotracheal intubation attempts, intubation via a double-lumen endobronchial tube, intubation before surgery, vasopressor or inotropic infusion before general anesthesia induction, and incomplete data were excluded from analysis. Univariate analysis was performed using logistic regression analysis, and the associated factors ($P < 0.2$) were subsequently selected for further multivariate logistic regression analysis. Factors associated with

post-induction hypotension in the fitted model were evaluated using receiver operating characteristic (ROC) curves.

Results: The median number of dialysis days was 2 day. Older age, especially over 65 years (p <0.001), long-term use of oral anti-diabetic drugs (p = 0.012), increased propofol dosage during induction (p =0.002), rapid sequence induction (p < 0.001), and emergency surgery (p = 0.002), were associated with post-induction hypotension. Differences in the number of days of dialysis or in weight compared to dry weight were not statistically significant. Post-induction hypotension in patients who underwent non-cardiac surgery under general anesthesia was 44.1% (95% [illegible]]: 40.28:47.98).

Conclusion: The risk of post-induction hypotension in patients with ESRD undergoing general anesthesia and emergency surgery was high in those aged over 65 years, patients on long-term oral anti-diabetic therapy, and cases involving rapid sequence induction or high doses of propofol.

Keywords: Post-induction hypotension, patients with ESRD, risk factors, non-cardiac surgery, general anesthesia

Introduction

Dialysis is a lifesaving measure for patients with end-stage renal disease (ESRD) to preserve their homeostasis. The extended lifespan achieved by dialysis has led to an increase in the need for surgery to treat underlying illnesses. In addition to having an adjusted all-cause death rate at least 10 times greater than that in the non-ESRD population, patients with ESRD are more likely to develop cardiovascular disease and other concomitant conditions [1,2].

The most common anesthetic approach for patients with ESRD is general anesthesia. However, general anesthesia

frequently lowers blood pressure through various mechanisms, including intravenous anesthesia vasodilation, which lowers systemic vascular resistance [3]. Thus, because inhalation agents inhibit the reflex heart rate response to blood pressure, they result in a greater decrease in blood pressure. Hypotension typically occurs during the post-induction period, but before surgical stimulation. Acute kidney injury, myocardial injury, and death are also associated with intraoperative hypotension [4]. Therefore, identifying risk factors for post-induction hypotension in patients with ESRD receiving general anesthesia is essential to reduce morbidity and mortality.

Prior research has been conducted in the general population. The risk factors for post-induction hypotension in surgical patients receiving general anesthesia have been systematically reviewed [5]. In seven trials, age and post-induction hypotension were found to be associated, with individuals aged 50 years or older being particularly prone to post-induction hypotension [6-12]. Moreover, five studies found a correlation between baseline MAP and post-induction hypotension [6,10-11,13-14], and one study found that baseline MAP < 70 mmHg was a significant risk factor [6]. Baseline SBP greater than 130 mmHg was a significant risk factor in one study, whereas three studies demonstrated a correlation between baseline SBP and post-induction hypotension [9]. The results for post-induction

hypotension and sex are contradictory [12,15]. According to two studies, post-induction hypotension is adversely correlated with weight [12,16]. According to one study, ASA physical status III-V is a significant risk factor [6]. Emergency surgery was substantially associated with post-induction hypotension in two trials [7,12]. In three studies, baseline blood volume was also a significant risk factor [14,16,17]. Angiotensin receptor blockers (ARBs) and angiotensin converting enzyme inhibitors (ACEIs) were found to be major risk factors for post-induction hypotension in two studies [10,15]. Premedication with a diuretic was a major risk factor for post-induction hypotension [17].

According to two trials, the use of propofol and increasing the dosage of fentanyl to induce general anesthesia were risk factors for post-induction hypotension [6,17]. A duration of less than 7 h between the dialysis session and the induction of general anesthesia caused hypotension in patients with ESRD during the post-anesthesia phase [18].

Therefore, this study was designed to determine the risk variables for post-induction hypotension in patients with ESRD undergoing general anesthesia.

Materials and Methods

Study design: Retrospective Cohort Study

The primary objective of this study was to identify the risk factors for hypotension after induction in patients with ESRD who underwent non-cardiac surgery under general anesthesia at Songklanagarind Hospital.

The secondary objective was to assess the incidence of post-induction hypotension in patients with ESRD who underwent non-cardiac surgery under general anesthesia at Songklanagarind hospital

Study population

This retrospective study included patients with ESRD (GFR < 15 mL/min/1.73 m^2) on chronic hemodialysis older than 18 years who underwent non-cardiac surgery under general anesthesia with endotracheal intubation between January 2012 and December 2022 at Songklanagarind Hospital. The study protocol was reviewed and approved by the Human Research Ethics Committee of the Faculty of Medicine at the Prince of Songkhla University (REC 66-290-8-1). Data were accessed for research purposes in 12/08/2023. Hospital records were obtained from the hospital information system (HIS), and patient consent was waived by the ethics committee because of the retrospective review of medical records. We excluded patients who underwent two or more attempts at endotracheal intubation, were intubated with a double-lumen endobronchial tube, had been intubated before surgery, received a vasopressor or inotropic infusion

before the induction of general anesthesia, or had incomplete data that affected the primary outcome (Incomplete medical records included systolic/diastolic blood pressure and heart rate during the pre-induction period and immediately, 5 min, 10 min, 15 min, and 20 min after endotracheal intubation). There were no informed consent or subject withdrawal criteria because of the retrospective nature of the study.

Variable definitions and data collection

Patient records were reviewed by the researchers and a trained anesthetic nurse, and data were extracted using forms and entered into Epidata before being exported to files for analysis.

Independent variables

Patient characteristics, including age, sex, body weight, height, BMI, dry weight, ASA physical status, comorbidity diseases (hypertension, diabetes mellitus, ischemic heart disease, significant arrhythmias, structural heart disease, cerebrovascular disease, peripheral vascular disease, hyperthyroidism, hypothyroidism, cancer, chronic obstructive pulmonary disease, and asthma), long-term medications (beta-blockers, calcium channel blockers, angiotensin converting enzyme inhibitors, angiotensin receptor blockers, diuretics, direct vasodilators, alpha blockers, central alpha 2 agonists, insulin, oral

antidiabetics, and thyroid therapy), preoperative laboratory (hematocrit, platelet, blood urea nitrogen, creatinine, sodium, potassium, chloride, bicarbonate, and calcium), premedication (beta-blockers, calcium channel blockers, angiotensin converting enzyme inhibitors, angiotensin receptor blockers, diuretics, direct vasodilators, alpha blockers, central alpha 2 agonists), days of dialysis before operation, and baseline blood pressure and heart rate in the ward (the average of maximum and minimum blood pressures before operation recorded in mL of mercury, the average of maximum and minimum heart rates before operation recorded in beats per minute), were reviewed. Surgical information included the type (elective or emergency) and site of surgery (intracranial, intrathoracic, intra-abdominal, superficial surgery, on extremities, ocular, E[illegible]T, laparoscopic, spinal, and others). Intraoperative variables included induction anesthetic medication (propofol, etomidate, midazolam, ketamine, thiopental, morphine, and fentanyl), rapid sequence induction technique, additional regional anesthesia (upper extremities, lower extremities, abdominal wall, and chest wall), inhalation agent (nitrous oxide, desflurane, and sevoflurane) intraoperative blood pressure and heart rate at T0 (immediately after arrival to the operation theatre), Ti (immediately after induction of anesthesia), T5 (after 5 min from induction of anesthesia), T10 (after 10 min from induction of anesthesia), T15 (after 15 min from induction of

anesthesia), and T20 (after 20 min from induction of anesthesia), and type and total dose of vasopressor (norepinephrine in µg, ephedrine in mg, adrenaline in µg)

Dependent variables (Outcomes)

The primary outcome was the identification of risk factors for post-induction hypotension in patients with ESRD who underwent noncardiac surgery under general anesthesia. Hypotension after induction of general anesthesia was defined as a > 30% decrease in MAP from baseline in the ward within 20 min after induction and the use of a vasopressor to treat hypotension. According to various definitions of hypotension after the induction of general anesthesia, we used the definition proposed by Bijker et al., which significantly increased the risk of postoperative stroke.

Sample size

The sample size was calculated from a formula according to a cohort study using the proportion of outcomes of interest in the exposed group and the proportion of outcomes in the unexposed group. No previous study has investigated the risk factors for post-induction hypotension in patients with ESRD undergoing general anesthesia. The sample size was calculated using data from a previous study finding risk factors for post-induction in patients

undergoing general anesthesia (6). The formula is as follows: Five percent significance and 80% were used.

$$n_{exposure} = \left[\frac{z_{1-\frac{\alpha}{2}} \sqrt{\bar{p}\bar{q}\left(1+\frac{1}{r}\right)} + z_{1-\beta}\sqrt{p_1 q_1 + \frac{p_2 q_2}{r}}}{\Delta} \right]^2$$

$$p_1 = P(outcome|exposure), \quad q_1 = 1 - p_1$$

$$p_2 = P(outcome|unexposure), \quad q_2 = 1 - p_2$$

$$p_1 = p_2 RR, \quad \bar{p} = \frac{p_1 + p_2 r}{1+r)}, \quad \bar{q} = 1 - \bar{p}$$

$$r = \frac{n_{unexposure}}{n_{exposure}}, \quad \Delta = p_1 - p_2$$

Sample size for the primary outcome: post-induction hypotension (analgesia with an increased dose of fentanyl)

Proportion of outcome occurring in exposure group (p1) = 0.103

Proportion of outcome occurring in non-exposure group (p2) = 0.042

Ratio (r) = 1.50

Sample size for exposure group = 228 and non-exposure group = 342

Total sample size = 570, included 10% dropout = 627

Sample size for primary outcome: factor of post-induction hypotension (aged 50 years or above)

Proportion of outcome occurring in the exposure group (p1) = 0.108

Proportion of outcome occurring in the non-exposure group (p2) = 0.02

Ratio (r) = 2.00

Sample size for the exposure group = 82 and non-exposure group = 164

Total sample size = 246, included 10% dropout = 271

Sample size for the primary outcome: factors of post-induction hypotension (use of propofol for induction)

Proportion of outcome occurring in the exposure group (p1) = 0.118

Proportion of outcome occurring in the non-exposure group (p2) = 0.03

Ratio (r) = 3.18

Sample size for the exposure group = 79 and non-exposure group = 251

Total sample size = 330, included 10% dropout = 363

Sample size for the primary outcome: pre-induction hypotension factors (baseline MAP < 70)

Proportion of outcome occurring in the exposure group (p1) = 0.211

Proportion of outcome occurring in the non-exposure group (p2) = 0.115

Ratio (r) = 2.63

Sample size for the exposure group = 152 and non-exposure group = 399

Total sample size = 551, included 10% dropout = 606

Therefore, the largest sample size for the primary outcome was from the factor “analgesia with increased dose fentanyl.” The total sample size was 571. The 10% dropout rate was 629.

Missing data

We used multiple imputations when a variable had ≤5% missing data, while variables with >5% missing data were excluded from the analysis.

Statistical analysis

For the baseline characteristics of patients in both groups, percentages were used to present the categorical variables, while mean and standard error were used for normally distributed continuous variables and median and IQR for non-normally distributed continuous variables. All parameters were analyzed using univariate analysis to explore the association between

independent factors and dependent outcomes. The chi-square test and Fisher’s exact test were used to test the association between categorical variables. Student’s t-test was used for normally distributed continuous variables, and the Wilcoxon rank sum test was used for non-normally distributed continuous variables. After univariate analysis, the independent variables that were clinically associated and those with a p-value < 0.2 were selected for further analysis using multivariate logistic regression. All predictive factors were entered into the model, and backward stepwise selection was used to determine the final model with the lowest AIC. The final model was a multivariate logistic regression analysis of delayed discharge. Odds ratios (ORs) and 95% confidence intervals (CIs) were used to determine association and significance. Statistical significance was set at P < 0.05. To test the accuracy of predictive factors for post-induction hypotension, receiver operating characteristic (ROC) was used for both models.

ROC Curve Analysis

Each group was analyzed using multivariate logistic regression. The model was tested using ROC curve analysis to plot the true-positive fraction (sensitivity) versus the false-negative fraction (1-specificity) across various cutoffs to generate an ROC curve. For the summarized entries, the effect of sensitivity and specificity area under the curve (AUC) was calculated for the

predicted discriminative ability between the post-induction hypotension and the post-induction normotension groups in the logistic regression model. As a result, excellent discrimination was defined by AUC > 0.9, good discrimination was defined by AUC 0.75–0.9, moderate or acceptable discrimination was defined by AUC > 0.6, and poor or random effect discrimination was defined by AUC <0.6. The model was modified by challenging each factor to determine the optimal AUC.

Results

Between 2012 and 2022, 714 patients with ESRD undergoing general anesthesia were identified in the Songklanagarind HIS. Patient inclusion is illustrated in the flowchart (Fig 1). The incidence of post-induction hypotension was 44.1% (95% CI: 40.2,47.9)

Fig 1. Flow chart of patient enrollment.

Table 1 shows patient characteristics according to post-induction hypotension and post-induction normotension in patients with ESRD undergoing general anesthesia. Patients with post-induction hypotension were more often older than 65 years (65–74 years 24.30% vs. 12.70%; > 74 years, 17.50% vs. 6.80%, P<0.001). Diabetes mellitus was more prevalent in the

hypotension group (42.90% vs. 28.20%, P<0.001); however, there was no difference in other underlying diseases. Sex, ASA of Anesthesiologists classification, body weight, BMI, and volume status did not differ between the groups. Patients with post-induction hypotension had higher insulin (15.00% vs. 7.61%, P=0.005) and oral antidiabetic drugs (9.29% vs. 2.82%, P<0.001) and lower calcium channel blockers (53.21% vs. 62.82%, P=0.018) and alpha blockers (20.71% vs. 29.86%, P=0.011) as long-term medications. Premedication with a central alpha 2 agonist resulted in lower post-induction hypotension rates (0.36% vs. 2.82%, P=0.028). Preoperative laboratory values showed lower bicarbonate (24.30(22.08,26.13) vs. 25.20(23.00,27.10), P<0.001) and calcium levels (9.70(9.00,10.30) vs. 10.00 (9.20,10.50), P=0.033) in the hypotension group, although the differences were not clinically significant.

Table 1. Demographics and characteristics data, comparing post-induction hypotension and normotension groups.

Characteristic	Normotension<br>)n = 355([a]	Hypotension<br>)n = 280([a]	P-value[b]
Sex			0.316

Characteristic	Normotension)n = 355([a]	Hypotension)n = 280([a]	P-value [b]
Male	184(51.8%)	133 (47.5%)	
Female	171(48.2%)	147(52.5%)	
Age group			<0.001
18-64 years	286 (80.6%)	163 (58.2%)	
65-74 years	45 (12.7%)	68 (24.3%)	
> 74 years	24 (6.8%)	49 (17.5%)	
ASA classification			0.349
3	351 (98.9%)	274 (97.9%)	
4	4 (1.1%)	6 (2.1%)	
Bodyweight (Kg)**	57 (50, 65)	57 (50, 65)	0.435
BMI (kg/m2) **	21.9 (19.9,24.9)	22.65 (20.3, 25.5)	0.271
Volume status			
Hypovolemia (Delta body weight <0)	10(2.82)	7(2.5)	1
Hypervolemia (Delta body weight >0)	345(97.18)	273(97.5)	
Underlying disease			
Hypertension	327 (92.1%)	263 (93.3%)	0.466

Characteristic	Normotension)n = 355([a]	Hypotension)n = 280([a]	P-value [b]
Diabetes mellitus	100 (28.2%)	120 (42.9%)	<0.001
Ischemic heart disease	39 (11%)	38 (13.57%)	0.385
Significant arrhythmia	26 (7.3%)	20 (7.1%)	1
Structural heart disease	15 (4.2%)	19 (6.79%)	0.213
Cerebrovascular disease	19 (5.35%)	24 (8.57%)	0.149
Peripheral vascular disease	7 (1.97%)	8 (2.86%)	0.641
Hyperthyroidism	3 (0.85%)	1 (0.36%)	0.634
Hypothyroidism	6 (1.69%)	6 (2.14%)	0.903
Cancer	6 (1.69%)	5 (1.79%)	1
COPD	4 (1.13%)	1 (0.36%)	0.390
Asthma	3 (0.85%)	5 (1.79%)	0.311
Long-term medication			
Beta-blockers	166 (46.76%)	143 (51.07%)	0.318
Calcium channel blockers	223 (62.82%)	149 (53.21%)	0.018
ACEIs	9 (2.54%)	7 (2.5%)	1
ARB	76 (21.41%)	62 (22.14%)	0.900

Characteristic	Normotension)n = 355([a]	Hypotension)n = 280([a]	P-value [b]
Diuretics	84 (23.66%)	81 (28.93%)	0.158
Direct vasodilators	144 (40.56%)	116 (41.43%)	0.890
Alpha blockers	106 (29.86%)	58 (20.71%)	0.011
Central alpha 2 agonists	14 (3.94%)	4 (1.43%)	0.098
Insulin	27 (7.61%)	42 (15%)	0.005
Oral antidiabetics	10 (2.82%)	26 (9.29%)	< 0.001
Thyroid therapy	5 (1.41%)	4 (1.43%)	1
PR-medication			
Beta-blockers	121 (34.08%)	108 (38.57%)	0.278
Calcium channel blockers	166 (46.76%)	118 (42.14%)	0.279
ACEIs	2 (0.56%)	3 (1.07%)	0.659
ARB	21 (5.92%)	19 (6.79%)	0.777
Diuretics	24 (6.76%)	29 (10.36%)	0.138
Direct vasodilators	91 (25.63%)	72 (25.71%)	1
Alpha blockers	22 (6.2%)	17 (6.07%)	1

Characteristic	Normotension<br>)n = 355([a]	Hypotension<br>)n = 280([a]	P-value[b]
Central alpha 2 agonists	10 (2.82%)	1 (0.36%)	0.028
Preoperative laboratory			
Hct (%)***	32.58 (5.81)	33.38 (6.17)	0.096
BUN (mg/dL)**	36.5 (24.55, 50.7)	34.65 (24.85, 46.45)	0.307
Cr (mg/dL)**	7.3 (5.3, 9.8)	6.8 (5, 9.1)	0.049
Bicarbonate (mmol/L)**	25.2 (23, 27.1)	24.3 (22.08, 26.13)	<0.001
Calcium (mmol/L)**	10 (9.2, 10.5)	9.7 (9, 10.3)	0.033
Intraoperative			
Propofol usage	351 (98.87%)	274 (97.86%)	0.349
Dose of propofol at induction**	120 (90,157.5)	140 (110, 180)	<0.001
Fentanyl usage	345 (97.18%)	271 (96.79%)	0.954

Characteristic	Normotension<br>)n = 355(ᵃ	Hypotension<br>)n = 280(ᵃ	P-value[b]
Dose of fentanyl at induction**	88.13 (30.43)	85.35 (27.28%)	0.240
Type of surgery (Emergency)	53 (14.93%)	30 (10.71%)	0.148
Rapid sequence induction technique	19 (5.35%)	38 (13.57%)	<0.001
Nitrous oxide used	4 (1.13%)	3 (1.07%)	1
Sevoflurane	257 (72.39%)	164 (58.57%)	<0.001
Desflurane	98(27.61%)	116(41.43%)	<0.001

[a]n (%), [b]Pearson's Chi-squared test; Wilcoxon rank sum test; Fisher's exact test

** Median (IQR), *** Mean(SD)

Intraoperative information between post-induction hypotension and post-induction normotension in patients with ESRD undergoing general anesthesia showed that post-induction hypotension was associated with a higher dosage of propofol as an induction agent (140(110,180) vs 120(90,157.50), P<0.001)

and sevoflurane as a volatile agent (58.57% vs 72.39%, P<0.001); however, no differences were found between the use of propofol as an induction agent, use of fentanyl as an analgesic agent, dosage of fentanyl, and use of nitrous oxide as a volatile agent.

Patients who underwent the rapid sequence induction technique had higher post-induction hypotension rates (13.57% vs. 5.35%, P<0.001). There was no significant difference between elective and emergency surgeries.

There were 18 (4.6%), 45 (11.5%), 57 (14.5%), 127 (32.4%), and 145 (37.0%) patients who had post-induction hypotension at T1, T5, T10, T15, and T20, respectively. The T20 time point presented the highest incidence of post-induction hypotension (37.0%).

Table 2. Hypotension events at any time points within 20 min after induction period according to day of dialysis.

Dialysis interval (days)	Time points				
	T1	T5	T10	T15	T20
1 day	8	21	31	53	60
2 days	8	18	22	54	57
At least 3 days	2	6	4	20	28

Total	18 (4.6%)	45 (11.5%)	57 (14.5%)	127 (32.4%)	145 (37.0%)

Fig 2 shows the median MAP at each time point. In the normotension group, it was 101 mmHg at baseline; immediately after arrival at the operating theater, it increased to 113 mmHg and then decreased from T0 to 107, 105, 104, 97, and 94 mmHg at T1, T5, T10, T15, and T20, respectively.

In the hypotension group, the median MAP was 99 mmHg at baseline; immediately after arrival at the operating theater, it increased to 104 mmHg and then decreased from T0 to 95, 90, 86, 75, and 71 mmHg at T1, T5, T10, T15, and T20, respectively. The lowest MAP was observed 20 min after intubation.

Fig 2. Aggregate plot median and interquartile range of mean arterial pressure during the induction and post-induction. * statistical significance.

Fig 3 shows the MAP from baseline, immediately after arrival at the operating room (T0) up to 20 min after endotracheal intubation, according to the number of dialysis days, as well as the aggregate plot median and IQR. There were no statistically

significant differences between the number of days in the dialysis group.

Fig 3. Aggregate plot median and interquartile range of mean arterial pressure during the induction and post-induction by the number of pre-op dialysis days.

Fig 4 shows the MAP from baseline up to 20 min after induction in patients who received more than 2.4 mg/kg propofol and more than 1.5 mcg/kg fentanyl and had significant hypotension (P=0.009).

Fig 4. Aggregate plot median and interquartile range of mean arterial pressure during the induction and post-induction by dose of propofol and fentanyl.

Table 3 summarizes the results from the multivariate logistic regression identifying risk factors for hypotension at a least one time point. These included age > 65 years (p <0.001), oral antidiabetic use as a long-term medication (p =0.012), dose of propofol (mg) (p =0.002), rapid sequence induction (p < 0.001), and type of surgery: emergency (p =0.002).

Table 3. Risk factors for hypotension at least one time point during 20 min after intubation, based on multivariate logistic regression.

Factors	Crude OR (95% CI)	Adj. OR (95% CI)	P (Wald's test)	p-value
Age group<br>Ref.= 18-64<br>65-74<br>75-88	 <br>2.65 (1.73,4.05)<br>3.71 (2.18,6.33)	 <br>2.04 (1.29, 3.24)<br>2.39 (1.31,4.35)	 <br>0.002<br>0.004	<0.001
Calcium channel blockers as long-term medication	1.51 (1.09,2.08)	1.34 (0.94,1.9)	0.101	0.101
Oral antidiabetics as long-term medication	3.57 (1.69,7.55)	2.83 (1.29,6.18)	0.009	0.007

Diuretic as premedication	8.01 (1.02,62.9)	5.61 (0.68,46.65)	0.11	0.052
Dose of propofol (mg)	0.992 (0.9889,0.9952)	0.9944 (0.9908,0.998)	0.002	0.002
Rapid sequence induction technique	2.80 (1.55,5.05)	3.83 (2.01,7.30)	< 0.001	< 0.001
Desflurane	0.54 (0.38,0.75)	0.69 (0.48,1)	0.049	0.050
Type of surgery: emergency	1.49 (0.91,2.44)	2.40 (1.37,4.19)	0.003	0.002
Volume status	1.58 (0.53,4.67)	2.58 (0.79,8.45)	0.119	0.109

Receiver operating characteristic (ROC) curve analysis revealed that the proposed model to discriminate patients with ESRD undergoing general anesthesia at risk of post-induction hypotension had an AUC of 0.732 (95% CI 0.692,0.771).

Fig 5. Area under the curve of the multivariate model.

Discussion

There are several definitions of post-induction hypotension. We selected a MAP decline of more than 30% from baseline for this study because it significantly increases the risk of stroke following surgery [19]. Based on this definition, this study had a higher incidence of post-induction hypotension than that reported by two other studies [8, 9] and a similar one to that in two other studies [10,14]. This study's decreased incidence compared to that in a study in older people [17] may be because the median age was lower (55 (18,88) years compared to 72.5 (68,78) years).

This study demonstrated that the T20 time point showed the highest incidence of post-induction hypotension. Another trial [6] reported that the incidence of hypotension was more frequent 5-10 min after induction, while another trial [17] indicated that the highest occurrence of hypotension was observed 15 min after induction. As all participants in our study had ESRD, which alters pharmacokinetics and pharmacodynamics by decreasing drug clearance, protein binding, and lean body mass, hypotension occurred at a delayed time point compared to that in other studies [20].

According to one study [6], ASA III-IV, age >50 years, pre-induction MAP <70 mmHg, propofol use as an induction

medication, and large fentanyl dosages (> 5 mcg/kg) were risk factors for hypotension. Increased age, pre-induction hypertension, and the presence of diabetes were shown to be predictive of post-induction hypotension in one study [9]. According to one study [7], pre-induction SBP, older age, and emergency surgery are independent risk factors for post-induction hypotension. A diuretic was found to be the preoperative medication in one trial [17], along with the degree of pre-induction hypertension and an increase in the dosage of fentanyl. Owing to the adoption of similar criteria for post-induction hypotension, these two studies [9,17] yielded similar results. According to our findings, there was no statistically significant difference between the pre-operative diuretic and pre-induction MAP hypertension. Conversely, our results indicate that age had increased. In their respective multivariate regression analyses, two earlier investigations [6, 21] also found a correlation for ages 50 and 66 years. Post-induction hypotension is more common in older adults, which may be explained by factors, such as baroreceptor dysfunction, reduced cardiac reserve, lower beta-adrenergic reactivity, and comorbidities [22].

Oral anti-diabetic medication was identified in this study as a long-term therapy linked to post-induction hypotension. A previous study reported a correlation between type II diabetes mellitus and post-induction hypotension [9]. Due to diabetic cardiovascular

autonomic neuropathy, the autonomic response to vasoconstriction and tachycardia does not completely offset the vasodilating effect of anesthesia, resulting in post-induction hypotension [24]. However, no previous study has linked oral anti-diabetic medications to post-induction hypotension. These findings suggest that oral anti-diabetic medications may increase the likelihood of post-induction hypotension in diabetic patients.

Consistent with an earlier study [6], higher propofol dosages for intraoperative management were associated with post-induction hypotension. Post-induction hypotension was associated with rapid sequence induction. This association may be explained by the administration of higher propofol dosages during rapid sequence induction. The mode of action of propofol, which inhibits the sympathetic nervous system and impairs the baroreflex regulatory mechanism, may help explain these findings [23].

According to our study, emergency surgery was associated with post-induction hypotension. In their individual multivariate regression analysis, two earlier trials [7,12] also found a correlation with emergency operation; thus, patients requiring emergency surgery, as opposed to elective surgery, are more likely to experience cardiocirculatory dysfunction due to their

underlying condition, particularly in patients with ESRD.

Limitations

This study has some limitations. First, blood pressure was intermittently recorded. Episodes of hypotension may have been missed during these intervals. Second, our study obtained measurements only up to 20 min after induction. The observed post-induction hypotension did not plateau within 20 min, suggesting that future studies should incorporate longer follow-up periods. Third, our study did not record the duration of the hypotension episodes. Fourth, while it is unlikely that surgical stimulation occurred 20 min after induction, mild stimulation may have occurred. If noxious stimuli had any effect during this period, they would be expected to decrease the incidence of post-induction hypotension. Fifth, the post-induction periods of intravenous fluids and inhalation agents were not recorded. Finally, as most dry weights cannot be accurately recalled from a patient's memory, it was not possible to link weight to post-induction hypotension. Therefore, a prospective approach should be developed for future research.

Conclusions

Increased age, long-term use of oral antidiabetic drugs, higher doses of propofol, rapid sequence induction, and emergency surgery were all risk factors associated with post-induction hypotension in patients with ESRD.

Acknowledgments

References

1. Brown JH, Hunt LP, Vites NP, et al. Comparative mortality from cardiovascular disease in patients with chronic renal failure. Nephrol Dial Transplant 1994;9:1136–42.
2. United States Renal Data System. 2016 USRDS annual data report: Epidemiology of kidney disease in the United States. End-stage renal disease (ESRD) in the United States, Volume 2. Bethesda, MD: National Institutes of Health, National Institute of Diabetes and Digestive and Kidney Diseases; 215–602.
3. Robinson BJ, Ebert TJ, O'Brien TJ, Colinco MD, Muzi M. Mechanisms whereby propofol mediates peripheral vasodilation in humans. Sympathoinhibition or direct vascular relaxation? Anesthesiology. 1997;86:64–72
4. Kouz K, Hoppe P, Briesenick L, Saugel B. Intraoperative hypotension: Pathophysiology, clinical relevance, and therapeutic approaches. Indian J Anaesth. 2020 :90-96.
5. Chen B, Pang QY, An R, Liu HL. A systematic review of risk factors for postinduction hypotension in surgical patients undergoing general anesthesia. Eur Rev Med Pharmacol Sci. 2021:7044-7050.
6. Reich DL, Hossain S, Krol M, Baez B, Patel P, Bernstein A, et al. Predictors of hypotension after induction of general anaesthesia. Anesth Analg 2005; 101: 622-628.

7. Sudfeld S, Brechnitz S, Wagner JY, Reese PC, Pinnshmidt HO, Reuter DA, et al. Post-induction hypotension and early intraoperative hypotension associated with general anaesthesia. Br J Anaesth 2017; 119: 57-64.
8. Choi MH, Chae JS, Lee HJ, Woo JH. Preanaesthesia ultrasonography of the subclavian/infracla- vicular axillary vein for predicting hypotension after inducing general anaesthesia. Eur J Anaesthesiol 2020; 37: 474-481.
9. Jor O, Maca J, Koutna J, Gemrotova M, Vymazal T, Litschmannova M, et al. Hypotension after induction of general anestehsia: occurrence, risk factors, and therapy. A prospective multicentre observational study. J Anesth 2018; 32: 673-680.
10. Okamura K, Nomura T, Mizuno Y, Miyashita T, Goto T. Pre-anesthetic ultrasonographic assess- ment of the internal jugular vein for prediction of hypotension during the induction of general an- aesthesia. J Anesth 2019; 33: 612-619.
11. Kaydu A, Guven DD, Gokcek E. Can ultrasonographic measurement of carotid intima-media thickness predict hypotension after induction of general anaesthesia? J Clin Monit Comput 2019; 33: 825-832.
12. Lin CS, Chang CC, Chiu JS, Lee YW, Lin JA, Mok MS, et al. Application of an artificial neural network to predict postinduction hypotension during general anaesthesia. Med Decis Making 2011; 31: 308-314.
13. Morley AP, Nalla BP, Vamadevan S, Strandvik G, Natarajan A, Prevost AT, et al. The influence of duration of fluid abstinence on hypotension during propofol induction. Anesth Analg 2010; 111: 1373-1377.
14. Zhang J, Critchley LAH. Inferior vena cava ultrasonography before general anaesthesia can predict hypotension after induction. Anesthesiology 2016; 124: 580-589.
15. Tarao K, Daimon M, Son K, Nakanishi K, Nakao T, Suwazono Y, et al. Risk factors including preoperative

echocardiographic parameters for post-induction hypotension in general anaesthe- sia. J Cardiol 2021; 78: 230-236.

16. Juri T, Suehiro K, Tsujimoto S, Kuwata S, Mukai A, Tanaka K, et al. Pre-anesthetic stroke volume variation can predict cardiac output decrease and hypotension during induction of general anaesthesia. J Clin Monit Comput 2018; 32: 415-422.

17. Sutthibenjakul, K., & Chatmongkolchart, S. (2021). Risk Factors for Postinduction Hypotension among Elderly Patients Undergoing Elective Non-Cardiac Surgery under General Anesthesia. JHSMC, 39(5), 391-400.

18. Deng J, Lenart J, Applegate RL. General anesthesia soon after dialysis may increase postoperative hypotension - A pilot study. Heart Lung Vessel. 2014:52-9.

19. Bijker JB, Persoon S, Peelen LM, Moons KG, Kalkman CJ, Kappelle LJ, et al. Intraoperative hypotension and perioperative ischemic stroke after general surgery. Anesthesiology 2012;116:658–64.

20. SladenRN Anesthetic considerations for the patient with renal failure. Anesthesiol Clin North Am 2000;18: 863–88210.1016

21. Cheung CC, Martyn A, Campbell N, Frost S, Gilbert K, Michota F, et al. Predictors of intraoperative hypotension and bradycardia. Am J Med 2015;128:532–8.

22. Lim BG, Lee IO. Anesthetic management of geriatric patients. Korean Journal of Anesthesiology 2020;73:8-29.

23. de Wit F, van Vliet AL, de Wilde RB, Jansen JR, Vuyk J, Aarts LP, et al. The effect of propofol on haemodynamics: cardiac output, venous return, mean systemic filling pressure, and vascular resistances. Br J Anaesth. 2016;116:784–9

24. Vinik AI, Ziegler D. Diabetic cardiovascular autonomic neuropathy. Endocrine Reviews. 2007;28:365-395. doi:10.1210/er.2007-0004.

Click here to access/download;Figure;Fig 1.docx

Fig 1.

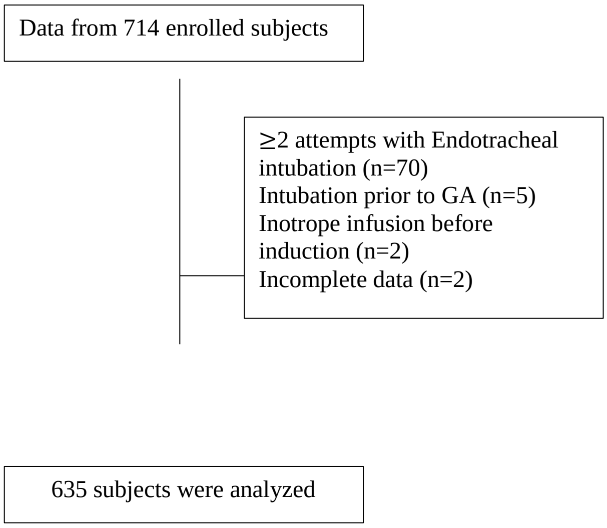

Commented [A1]: "Figure files are uploaded separately."
I have moved all figures to a temporary Word file so you may prepare them for submission.

"File Naming for Figures:
• Figure files should be saved as "Fig1.tif", "Fig2.eps", etc.
• Acceptable file formats for figures are ".tif", ".tiff", and ".eps"
• Figures should be uploaded separately as individual files."

Click here to access/download;Figure;Fig 2.docx

Fig 2.

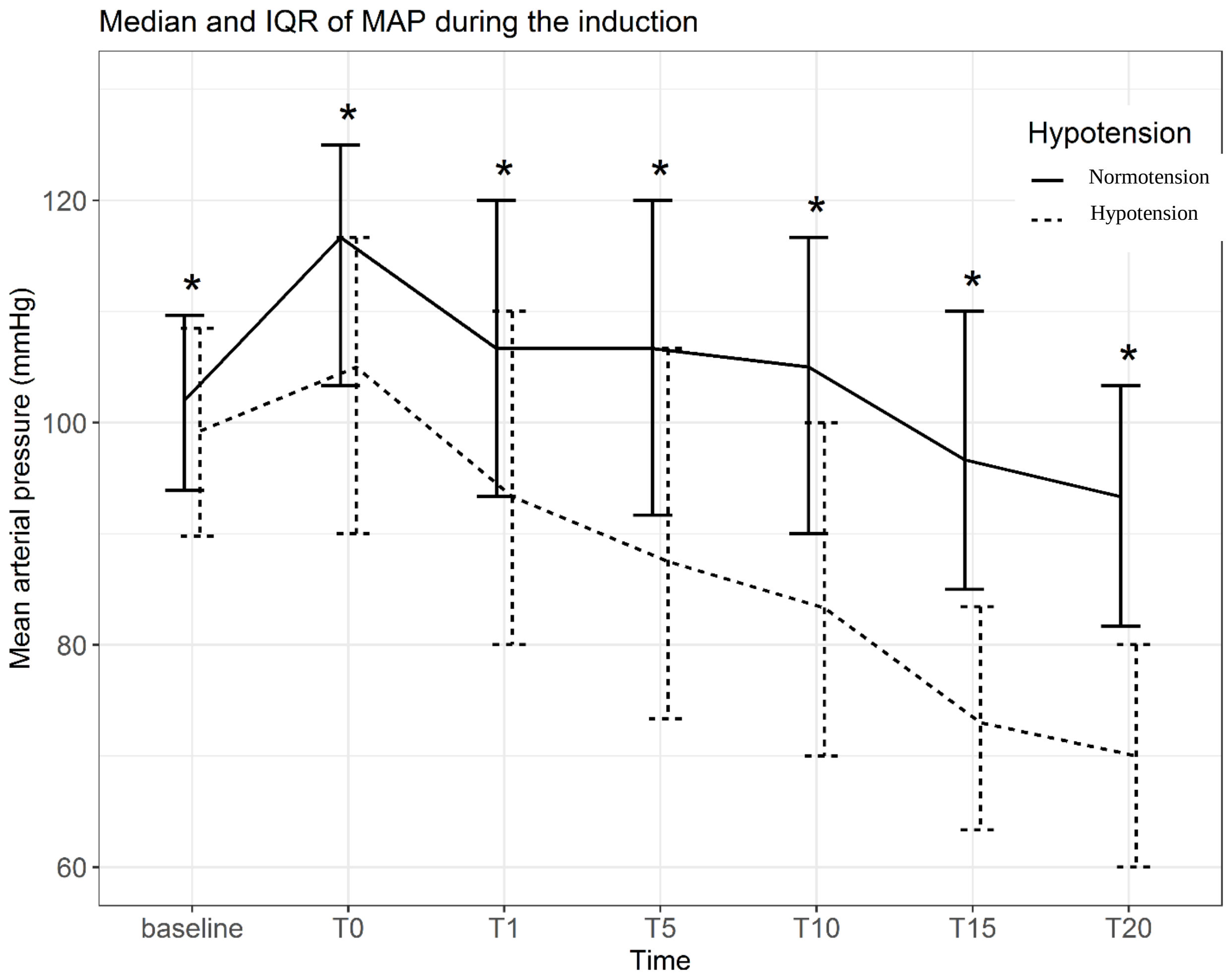

Click here to access/download;Figure;Fig 3.docx

Fig 3.

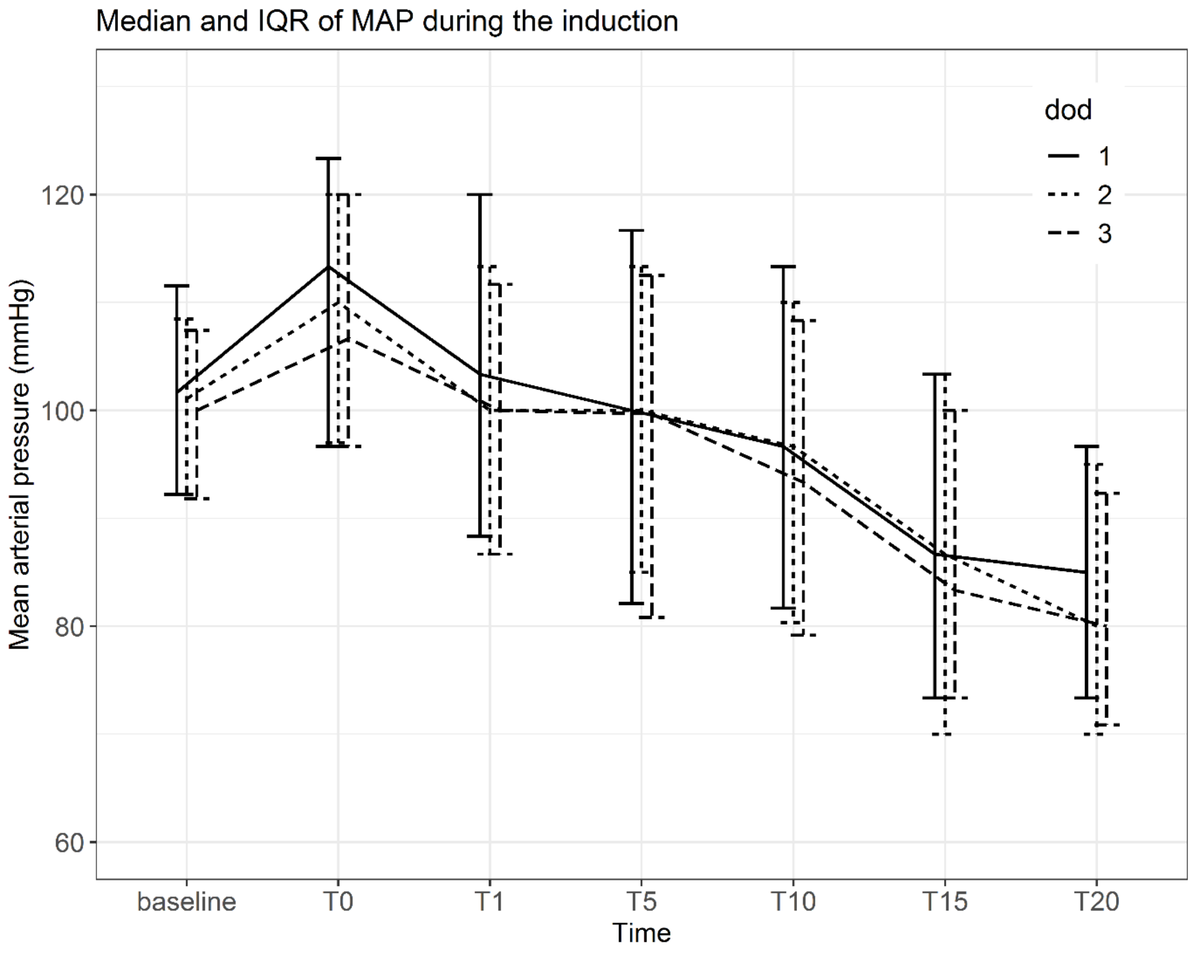

Click here to access/download;Figure;Fig 4.docx

Fig 4.

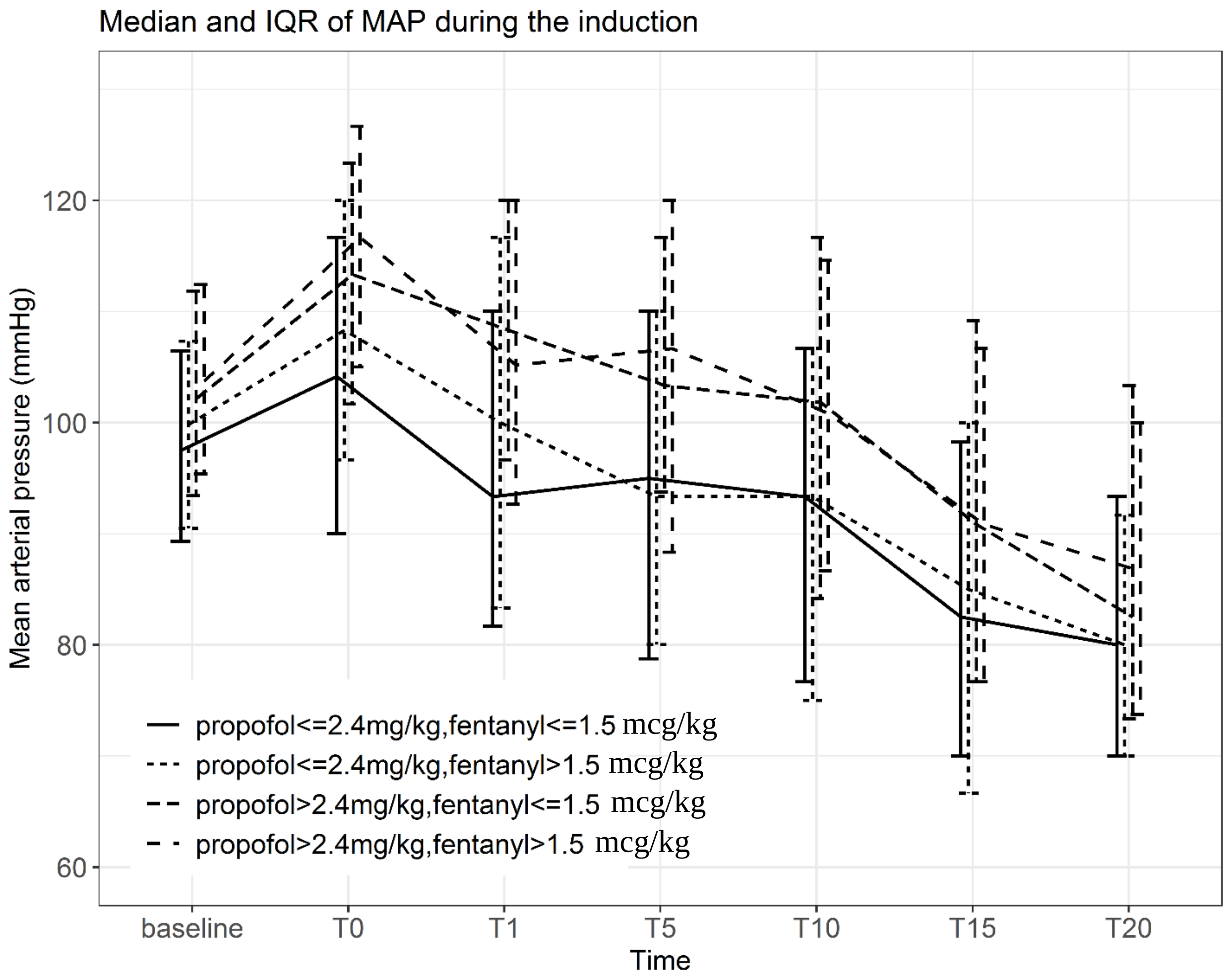

Click here to access/download;Figure;Fig 5.docx

Fig 5.

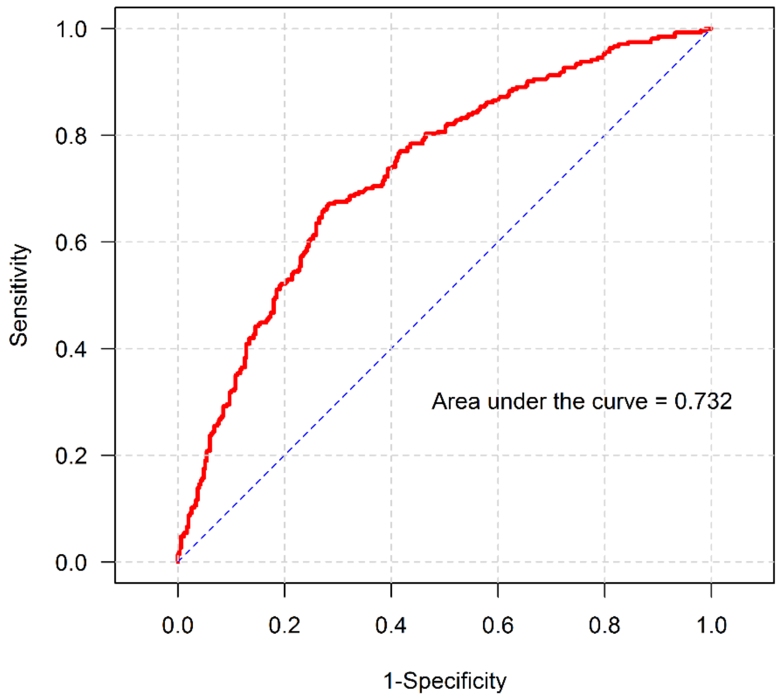

Supplement: Attachment — Submitted filename: PONE-D-25-50403.pdf [file pone.0350083.s002.pdf]
