# Supplementary material for: Risk factors for post-induction hypotension in patients with end-stage renal disease undergoing general anesthesia
Source: PLoS One. 2026 May 29;21(5):e0350083. doi: 10.1371/journal.pone.0350083 (PMC13221025; doi:10.1371/journal.pone.0350083)
Supplement: Attachment — Submitted filename: Response to reviewer.docx [file pone.0350083.s004.docx]

**Response to reviewer**

**Reviewer #1**

Thank you for the opportunity to revise our manuscript. We have carefully addressed all comments and suggestions provided in the PDF file. All changes have been incorporated into the revised manuscript, and the corresponding modifications are clearly highlighted for your convenience.

**Reviewer #2**

**-While the manuscript addresses relevant points, the statistical methodology is not rigorous and needs to be revised.**

Answer Thank you for this important comment. We agree that rigorous statistical methodology is essential to support the validity of our findings. In response, we have carefully revised and strengthened the statistical analysis of the manuscript.

Specifically, we clarified the statistical approach by explicitly describing the rationale for variable selection, including the use of univariate screening (P < 0.20) combined with clinical relevance before entry into the multivariate logistic regression model. Multicollinearity among covariates was assessed prior to model construction. The final multivariate model was obtained using backward stepwise selection based on the lowest Akaike Information Criterion (AIC).

We also revised the handling of missing data by clearly specifying the use of multiple imputation for variables with ≤5% missing values, while excluding variables with greater degrees of missingness from the analysis. Model performance was evaluated using receiver operating characteristic (ROC) curve analysis, and the discriminative ability of the final model is now explicitly reported using the area under the curve (AUC) with 95% confidence intervals.

In addition, we revised the Statistical Analysis and Results sections to improve clarity, consistency, and transparency, and we ensured that adjusted odds ratios with 95% confidence intervals are consistently reported. These revisions are intended to improve the rigor, reproducibility, and interpretability of the statistical methodology.

We believe that these changes have substantially strengthened the statistical robustness of the study and addressed the reviewer’s concern.

**-Results are presented in a disorganized way.
-Flow between sections is sometimes unclear. Several ideas are presented without strong connections to available evidence, which makes the argument feel weak. Improving the logical connections between concepts and better integrating the sections would enhance readability and coherence.**

Answer We acknowledge that the initial presentation of our results and discussion lacked optimal organization and clarity. In response to this comment, we have thoroughly revised the manuscript to improve the structure, logical flow, and coherence between sections.

Specifically, we have reorganized the Results section to follow a clear and systematic sequence, beginning with participant characteristics, followed by primary outcomes, secondary outcomes, and multivariable analyses. We have also ensured that all tables and figures are presented in a logical order and are clearly referenced in the text.

**-There should be consistency in the presentation of the results. Why day of dialysis is not included in table 1? Why is it presented separately in table 2? Why some factors and time interaction are presented as figures and other factors no.**

Answer We acknowledge that the initial presentation of our results lacked optimal consistency. The variable “days of dialysis before surgery” was originally analyzed primarily in relation to temporal changes in mean arterial pressure after induction; therefore, it was presented separately in Table 2 and Figure 3 to demonstrate its association with hypotension events over time. However, we agree that this variable should also be included in the baseline characteristics.

We have now revised Table 1 to include days of dialysis as part of the demographic and preoperative characteristics to ensure comprehensive baseline reporting.

Regarding the presentation of time–factor interactions, selected variables (such as days of dialysis and anesthetic drug dosage) were illustrated as figures to demonstrate their dynamic relationship with blood pressure changes over time, while other variables were presented in tabular form due to their static nature or lack of significant interaction with time. We acknowledge that this approach was not sufficiently explained in the original manuscript.

**-Time interactions with hypotension and with different predictors are not listed as secondary outcomes.**

Answer Regarding time interactions, we acknowledge that interactions between time and hypotension with different predictors were not explicitly listed as secondary outcomes in the original manuscript. We have now clarified in the Methods section that time-related changes in mean arterial pressure according to key predictors were explored as secondary analyses and are presented in the corresponding figures. We have also revised the Results section to explicitly describe these time–predictor interactions.

**-The sample size calculation should take into account all the predictors at the same time.**

Answer With respect to the sample size calculation, we agree that ideally it should account for multiple predictors simultaneously in a multivariable model. However, due to the lack of prior studies in patients with ESRD and post-induction hypotension, we based our sample size estimation on the strongest reported individual predictors from previous literature. We therefore calculated the required sample size using several key risk factors and selected the largest estimated sample size to ensure adequate statistical power. This approach has now been clarified in the Sample Size section as a pragmatic strategy for this retrospective study.

**-The quality of the English needs improvement. Some errors need to be corrected. Example: mm Hg rather than mL of mercury.**

Answer We have carefully revised the manuscript to improve the overall language quality and correct grammatical, typographical, and technical errors.

**-Did the authors follow the Strobe guidelines?**

Answer Yes, this study was conducted and reported in accordance with the STROBE (Strengthening the Reporting of Observational Studies in Epidemiology) guidelines for observational studies. We have reviewed the STROBE checklist and ensured that all relevant items are appropriately addressed in the manuscript, including study design, participant selection, data collection, statistical analysis, and reporting of results.

**-It is not clear whether the authors followed their definition of hypotension which has two components: 30% decrease AND vasopressor use. There is no mention of vasopressor use in the results section and throughout the manuscript.**

Answer We confirm that our definition of post-induction hypotension required both components: (1) a decrease in mean arterial pressure of more than 30% from baseline and (2) the administration of vasopressor therapy within 20 minutes after induction. Only patients who fulfilled both criteria were classified as having post-induction hypotension and included in the hypotension group for analysis.

Vasopressor use was systematically recorded in our case record forms and hospital information system, including norepinephrine, ephedrine, and adrenaline administered during the post-induction period. However, we acknowledge that this information was not clearly described in the Results section of the original manuscript.

We have now revised the Methods and Results sections to explicitly state that all hypotension events analyzed in this study met both the blood pressure criterion and required vasopressor treatment, and we have added a description of vasopressor use in the Results section to improve clarity.

**-I am not sure of the association between oral antidiabetics and hypotension. What about insulin? It is probable that the association is between diabetes and hypotension rather than with the antidiabetic drugs.**

Answer We agree that the observed association is more likely related to diabetes itself rather than a direct pharmacological effect of oral antidiabetic agents. Diabetes, particularly in patients with long-standing disease, is frequently associated with cardiovascular autonomic neuropathy, which impairs compensatory vasoconstriction and heart rate responses during anesthesia induction. This mechanism may predispose patients to post-induction hypotension.

In our study, both insulin and oral antidiabetic use were more prevalent in the hypotension group in univariate analysis. However, after multivariate adjustment, only oral antidiabetic use remained statistically significant. We interpret this finding as a surrogate marker of underlying diabetes severity and chronicity rather than a causal effect of the medication itself.

We have revised the Discussion section to clarify that the association likely reflects the impact of diabetes-related autonomic dysfunction rather than the direct effect of antidiabetic drugs, and we have acknowledged this as a limitation of our retrospective design.

**-Doses of propofol and fentanyl should be reported per body weight.
-Units should be added to the tables, such as mg for propofol and microgram for fentanyl.**

Answer We have revised the manuscript as suggested, as shown in the attached file.

Reviewer #3: This study addresses post-induction hypotension in patients with end-stage renal failure. Still, it does not meet the scientific depth, novelty, and contribution to the literature required for publication in a high-impact journal. The main findings of the study reiterate previously identified and well-known risk factors in the existing literature; it does not offer a new conceptual framework, methodological approach, or original contribution that would change clinical practice. For these reasons, the study is unsuitable for publication.

**1. The main risk factors identified in the study have been previously demonstrated in the literature. The findings do not offer a new inference that advances existing knowledge or changes clinical practice.**

Answer We acknowledge that several risk factors identified in our study, such as advanced age, propofol dosage, and emergency surgery, have been reported in previous studies. However, we respectfully believe that our study provides meaningful and clinically relevant contributions to the existing literature for several reasons.

First, to our knowledge, this is one of the largest single-center studies focusing specifically on patients with end-stage renal disease undergoing non-cardiac surgery under general anesthesia. Most previous studies have evaluated heterogeneous surgical populations, whereas data focusing exclusively on ESRD patients remain limited. Given the unique physiological characteristics of ESRD, including altered pharmacokinetics, autonomic dysfunction, and volume status variability, risk factors identified in the general population cannot be directly extrapolated to this vulnerable group.

Second, our study provides a comprehensive evaluation of both patient-related and anesthesia-related factors in ESRD patients, including dialysis timing, dry weight status, detailed medication profiles, and time-dependent blood pressure changes after induction. The finding that dialysis interval and volume status were not significantly associated with post-induction hypotension challenges common clinical assumptions and provides important evidence for perioperative management in this population.

Third, we applied a clinically relevant and stringent definition of post-induction hypotension that combined both hemodynamic criteria and vasopressor requirement, thereby identifying hypotensive episodes with clear therapeutic and prognostic significance. This approach improves the clinical applicability of our findings.

Fourth, our analysis highlights the association between long-term oral antidiabetic use and post-induction hypotension in ESRD patients, which has not been consistently reported in previous studies. We interpret this finding as a surrogate marker of diabetic autonomic dysfunction, offering additional insight into risk stratification beyond conventional comorbidity classification.

Finally, the moderate discriminatory performance of our predictive model (AUC = 0.732) suggests that commonly available clinical variables can be used to identify high-risk ESRD patients preoperatively. This may assist clinicians in optimizing induction strategies and implementing targeted hemodynamic monitoring and preventive measures.

**2. The introduction is unnecessarily long and summarizes numerous studies sequentially. Many details in this section serve as a literature review rather than clarifying the study's original hypothesis.**

Answer We have substantially revised the Introduction to improve its focus and clarity.

**3. Some issues that require in-depth analysis in the discussion section are addressed superficially in the introduction and evaluated only to a limited extent in the discussion section. There is no logical hierarchy or continuity of focus between the introduction and discussion sections. The discussion section primarily repeats the results.**

Answer We have substantially revised the Discussion section to improve its depth, structure, and coherence with the Introduction. Specifically, we reorganized the Discussion to establish a clear logical hierarchy, beginning with the principal findings and followed by focused sections addressing the underlying mechanisms and clinical relevance of each major risk factor. We expanded the interpretation of key results, including the roles of age, diabetes-related autonomic dysfunction, anesthetic techniques, and emergency surgery, and linked these findings more explicitly to the study rationale presented in the Introduction.

**4. A significant portion of the references is 10–20 years old.
Current, high-impact studies published within the last 5 years, particularly on topics such as intraoperative hypotension, hemodynamic management, and anesthesia in high-risk patient groups, have not been sufficiently utilized.**

Answer We acknowledge that a substantial proportion of the original references were older and did not sufficiently reflect recent advances in perioperative hemodynamic management.

In response, we have thoroughly updated the reference list by incorporating recent, high-impact studies published within the last five years, particularly those focusing on intraoperative hypotension, perioperative hemodynamic optimization, and anesthesia management in high-risk patient populations. These newly added references have been integrated into the Introduction and Discussion sections to strengthen the scientific context and support the interpretation of our findings.

**5. The text contains repetitive sentences and long paragraphs; the language should be simplified. Some figures and tables are redundant and could be simplified.**

Answer We have carefully revised the manuscript to improve clarity, readability, and conciseness. Specifically, we simplified the language throughout the text, shortened long paragraphs, and removed repetitive sentences. Several sections, including the Abstract, Methods, and Results, were condensed to present key information more clearly and directly.
