# Supplementary material for: Risk factors for post-induction hypotension in patients with end-stage renal disease undergoing general anesthesia
Source: PLoS One. 2026 May 29;21(5):e0350083. doi: 10.1371/journal.pone.0350083 (PMC13221025; doi:10.1371/journal.pone.0350083)
Supplement: Attachment — Submitted filename: response_to_reviewer_auresp_2.docx [file pone.0350083.s005.docx]

**RESPONSE TO REVIEWERS**

Manuscript Title: Risk factors for post-induction hypotension in patients with end-stage renal disease undergoing general anesthesia

We sincerely thank the Editor and the Reviewers for their constructive comments. We have carefully revised the manuscript and addressed all concerns. All changes have been incorporated into the revised manuscript.

**Reviewer #1**

**Comment:** All comments have been addressed.

**Response:**
We sincerely thank the reviewer for the positive evaluation and for acknowledging that all concerns have been adequately addressed.

**Reviewer #3**

We thank the reviewer for the detailed and critical assessment. We have carefully considered all comments and substantially revised the manuscript to improve its scientific rigor, clarity, and contribution.

**Comment 1:**

The main risk factors identified in the study have been previously demonstrated. The findings do not offer new insights or change clinical practice.

**Response:**
We agree that several identified risk factors have been reported in previous studies. However, we would like to emphasize that our study specifically focuses on **patients with end-stage renal disease (ESRD)**, a population with distinct pathophysiological characteristics that are often underrepresented in the literature.

To address this concern, we have:

- Clearly emphasized the **population-specific contribution** of our findings
- Highlighted the **high incidence (44.1%)** of hypotension in ESRD patients
- Added discussion on the **delayed onset (peak at 20 minutes)** of hypotension
- Clarified that **dialysis-related factors were not associated**, which contrasts with common assumptions

These additions are now explicitly stated in the Abstract and Discussion sections.

**Comment 2:**

The introduction is unnecessarily long and resembles a literature review.

**Response:**
We agree and have substantially revised the Introduction. Specifically, we have:

- Reduced the length by removing redundant literature summaries
- Focused on establishing the **knowledge gap** and study rationale
- Improved logical flow leading to the study objective

**Comment 3:**

The discussion lacks depth and mainly repeats results. There is no logical continuity.

**Response:**
We appreciate this important comment and have comprehensively rewritten the Discussion section. Changes include:

- Adding a **"What this study adds"** subsection
- Expanding mechanistic explanations (autonomic dysfunction, pharmacodynamics)
- Improving integration with current literature
- Ensuring logical progression from findings → interpretation → clinical implications

**Comment 4:**

References are outdated; recent high-impact studies are lacking.

**Response:**
We have updated the reference list by incorporating multiple recent studies (within the last 5 years).

**Comment 5:**

Language issues, repetition, and redundant figures/tables.

**Response:**
We have thoroughly revised the manuscript for language and clarity. Specifically:

- Rewritten the manuscript in **clear, concise academic English**
- Removed repetitive sentences and shortened long paragraphs
- Simplified figures and tables to eliminate redundancy
